# Microfluidics-Assisted Three-Dimensional Confinement of Cholesteric Liquid Crystals for Sensing Applications

**DOI:** 10.3390/mi17020244

**Published:** 2026-02-13

**Authors:** Jiamei Chen, Xinyi Feng, Jiaying Huang, Xinyi Li, Shijian Huang, Zongbing Wu, Lvqin Qiu, Liping Cao, Qi Liang, Xiaoyan Li

**Affiliations:** 1 The Seventh Clinical College of Guangzhou University of Chinese Medicine, Guangzhou University of Chinese Medicine, Shenzhen 518133, China; chenjiamei90@gzucm.edu.cn (J.C.);; 2 Guangdong Basic Research Center of Excellence for Structure and Fundamental Interactions of Matter, Guangdong Provincial Key Laboratory of Nanophotonic Functional Materials and Devices, School of Optoelectronic Science and Engineering, South China Normal University, Guangzhou 510006, China; 3 School of Medical Technology and Information Engineering, Zhejiang Chinese Medical University, Hangzhou 310000, China

**Keywords:** microfluidics, cholesteric liquid crystals, 3D spherical confinement, sensors

## Abstract

As a class of self-organized soft matter systems merging fluidic mobility with long-range molecular order, cholesteric liquid crystals (CLCs) possess immense potential for the development of high-sensitivity, visually tractable flexible sensors. Leveraging their unique helical superstructures and stimuli-responsive photonic bandgaps, CLCs can transduce subtle physical or chemical perturbations into discernible optical signatures, such as Bragg reflection shifts or mesomorphic textural transitions. Nonetheless, the intrinsic fluidity of CLCs often compromises their structural integrity, while conventional one-dimensional (1D) or two-dimensional (2D) confinement geometries exhibit pronounced angular dependence, significantly constraining their detection precision in complex environments. Recently, microfluidic technology has emerged as a pivotal paradigm for achieving sophisticated three-dimensional (3D) spatial confinement of CLCs through the precise manipulation of microscale fluid volumes. This review systematically delineates recent advancements in microfluidics-enabled CLC sensors. Initially, the fundamental self-assembly principles and optical properties of CLCs are introduced, emphasizing the unique advantages of 3D spherical confinement in mitigating angular sensitivity and intensifying interfacial interactions. Subsequently, the primary sensing mechanisms are bifurcated into bulk-driven sensing via pitch modulation and interface-driven sensing via topological configuration transitions. We then detail the microfluidic-based fabrication strategies and engineering protocols for diverse 3D architectures, including monodisperse/multiphase droplets, microcapsules, shells, and Janus structures. Building upon these structural frameworks, current sensing applications in physical (temperature, strain/stress), chemical (volatile organic compounds, ions, pH), and biological (biomarkers, pathogens) detection are evaluated. Lastly, in light of persistent challenges, such as intricate signal interpretation and limited robustness in complex matrices, we propose future research trajectories, encompassing the co-optimization of geometric parameters (size and curvature), artificial intelligence-enhanced automated diagnostics, and multi-field-coupled intelligent integration. This work seeks to provide a comprehensive roadmap for the design of next-generation, high-performance, and portable liquid-state photonic sensing platforms.

## 1. Introduction

Sensors, as devices or systems that detect and convert physical or chemical quantities into measurable signals, are indispensable in modern society [1]. Driven by advancing technology and growing demands, sensor development is progressing toward greater intelligence, miniaturization, and integration. This evolution is essential for enabling flexible, compact, and biocompatible devices in fields such as wearable electronics [2,3], functional textiles [4,5], implantable medical devices [6], and soft robotics [7]. In particular, the performance and applicability of a sensor are fundamentally governed by its sensing element, specifically by its morphology, dimensions, and material properties. In recent years, soft matter has emerged as an ideal sensing material for constructing next-generation flexible sensors, owing to their inherent flexibility, stretchability, and excellent biocompatibility [8]. These materials are a class of condensed matter substances (e.g., gels and polymers) that lie between ideal solids and fluids and exhibit pronounced responses to external stimuli. Soft matter-based sensors can conform closely to complex or irregular surfaces (human skin or tissue) [9], and offer high structural tunability for diverse device design. Therefore, exploring how to further utilize the dynamic response of soft matter and enhance its sensing performance has emerged as a major research direction.

Cholesteric liquid crystals (CLCs) are self-organized soft matter phases, typically formed by doping nematic liquid crystals (NLC) with chiral dopants. This doping induces a periodic helical superstructure via intermolecular chiral induction, which in turn gives rise to a unique long-range ordered structure [10,11]. This structure gives rise to a photonic bandgap in the visible or near-infrared region, and exhibits a pronounced responsiveness to external stimuli. Therefore, CLCs can sensitively detect minute physical or chemical perturbations, including temperature [12,13], stress [14,15], or specific molecular adsorption [16,17,18] and directly convert them into alterations such as wavelength shift or changes in microscopic configuration and macroscopic texture [19,20]. This inherent capability for signal amplification and visualization endows CLC-based sensors with distinct advantages, including high sensitivity, rapid response, visual readout, and excellent reversibility.

However, the inherent fluidity and structural instability of CLCs severely limit their direct fabrication into stable and practical devices. To overcome these limitations, such as enhancing mechanical stability, enabling patterned integration, and protecting the sensitive structure, physical or chemical confinement is essential. Based on the geometry of confinement, the strategies are broadly classified into three types: one-dimensional (1D) confinement (e.g., in planar cells or thin films), two-dimensional (2D) confinement (e.g., within fibers or microchannels), and three-dimensional (3D) confinement (e.g., in droplets or microcapsules). Among these, 3D confinement can be further categorized by shape, including non-spherical geometries (such as cubes or cylinders) and spherical geometries, most commonly as CLC droplets. Compared to other geometries, spherical confinement is particularly promising for sensing because its spherical symmetry significantly reduces angular sensitivity in optical responses and provides a high specific surface area to enhance interfacial interactions. Given these advantages, microfluidic technology becomes crucial for the precise fabrication of monodisperse, 3D-confined CLC architectures with tailored structures. This review first introduces the fundamental properties and sensing mechanisms of CLCs, the necessity of confinement, and the limitations of low-dimensional approaches, with a focus on microfluidic technology as the key to achieving ideal 3D confinement. It subsequently summarizes recent advances in sensing applications using microfluidics-confined CLCs and discusses current challenges and future perspectives.

## 2. CLCs: From Molecular Self-Assembly to Sensing Principles

### 2.1. Helical Structure and Optical Properties

**Figure 1:**
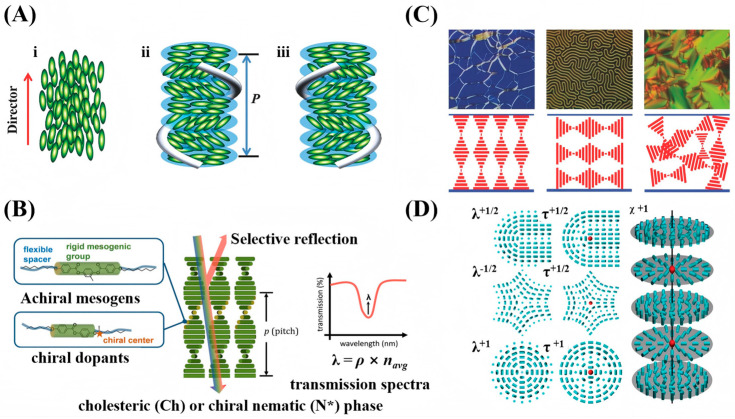
Characteristics of CLCs: (**A**) helical structure, (**i**) nematic and (**ii**,**iii**) cholesteric (chiral nematic) LC phases [19]; (**B**) selective reflection of the chiral nematic (N*). The asterisk denotes chirality. [23]; (**C**) optical textures and helical axis orientation [19]; (**D**) topological defects [26]. Adapted with permission from Ref. [19], Copyright 2018 John Wiley & Sons—Books. Adapted with permission from Ref. [26]. Copyright 2012 Royal Society of Chemistry.

CLCs represent an important phase within liquid crystalline materials [21,22]. Their structure is intermediate between that of isotropic liquids and crystalline solids, combining liquid-like fluidity with crystal-like molecular order. Typically, CLCs are formed either by doping a nematic host with chiral dopants or by using chiral mesogens that self-assemble into the cholesteric phase directly [11]. Depending on their chiral handedness, they are classified as left- or right-handed. Their most distinctive feature is the periodic helical superstructure. In this structure, rod-like molecules within local quasi-layers share a common orientation, defining a director. This director rotates continuously from layer to layer along the helical axis, and the distance over which it completes a full 360° turn is known as the helical pitch (*P*) [11,19], as shown in Figure 1A. The periodic helical arrangement makes CLCs a 1D photonic crystal, with a refractive index periodically modulated along the helical axis to form a photonic bandgap, as depicted in Figure 1B. Circularly polarized light whose handedness matches that of the helical structure is selectively reflected within the bandgap, while all other light is transmitted. When the reflected wavelength lies in the visible spectrum, structural color emerges. The central reflection wavelength (λ) can be approximated by a modified Bragg’s law [23,24]:

$$\lambda =n_{av}Pcos\theta$$

Here, λ is the reflected wavelength, P is the helical pitch, θ is the angle between the incident light and the helical axis, and $n_{av}$ is the average refractive index.

The pitch determines the position of the photonic bandgap, while the orientation of the helical axis dictates how light interacts with it. This orientation is primarily determined by surface anchoring when CLCs are confined within a liquid crystal cell (with two parallel substrates), resulting in three key textures: the Grandjean texture (axis perpendicular, selective reflection), fingerprint texture (axis in-plane, periodic stripes), and focal conic texture (axis random, strong scattering) [19], as shown in Figure 1C. Changes in these textures and their corresponding optical properties (color, stripes, transparency) can be utilized for sensing applications.

When confined within spherical geometries, CLCs exhibit different configurations. The equilibrium configuration arises from the interplay of elasticity, chirality, and interfacial anchoring effects [25,26]. When elasticity and chirality are fixed, the anchoring condition plays the key role. Two fundamental types are most commonly observed: under planar anchoring, the CLCs molecules align parallel to the interface, and the helical axis extends radially from the center of the droplet outward, forming a radially aligned configuration. In contrast, under homeotropic anchoring, the molecules orient perpendicular to the interface at the surface, while the helical axis tends to align tangentially along the interface. This type of structure is often accompanied by double-spiral defects or focal conic textures, macroscopically appearing as fingerprint-like or polydomain patterns with strong light-scattering properties. Furthermore, through external stimuli such as light or heat, or material treatments like polymer stabilization or chirality inversion, more complex dynamic topological structures including point defects, line defects, and even ring defects can be induced [26], as shown in Figure 1D. Such configuration transitions, driven by changes in interfacial anchoring, can produce extremely pronounced optical signal changes, making them highly suitable for high-sensitivity sensing.

### 2.2. Principles of Reduced Angular Dependence in 3D Spherically Confined CLCs

**Figure 2:**
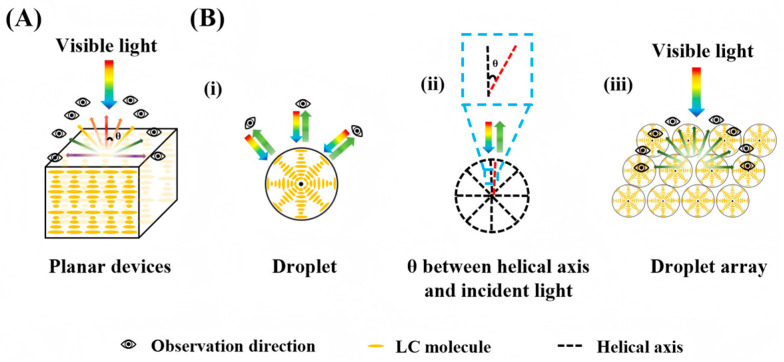
Schematic diagram of the CLC molecular alignment and the angular sensitivity of the droplet array. (**A**) Molecular alignment in a planar device; (**B**) Molecular alignment in a droplet. (**i**) Schematic of a single CLC droplet reducing angular dependence. (**ii**) Close-up view: Helical axis of a single CLC droplet is nearly parallel to incident light, with a small angle θ. (**iii**) In a droplet array, local helical axes always exist parallel to the incident light, significantly reducing angular dependence.

Achieving ideal reduction in angular sensitivity in traditional CLC structures remains a significant challenge. In conventional planar configurations or when encapsulated within optical fibers or capillaries, the photonic bandgap is typically confined strictly to the thickness direction or along the long axis. This 1D bandgap structure results in a strong angular dependence on the reflection wavelength: according to Bragg’s law, an increase in the viewing angle induces a significant blue shift (i.e., a decrease in wavelength) of the selective reflection, as illustrated in Figure 2A. This inherent angular sensitivity severely restricts the potential of CLCs in applications such as wide-angle displays, omnidirectional lasers, and precision sensing. In contrast, encapsulating CLCs into 3D geometries—such as droplets, microspheres, shells, or microcapsules via microfluidics can reduce its angular sensitivity. Under planar anchoring conditions, symmetrically 3D-confined CLC can self-assemble into a “Bragg-onion” superstructure with radially oriented helical axes, as shown in Figure 2B. This structure is essentially a 3D photonic crystal, in which the helical axes are arranged radially outward from the center of the sphere, covering all spatial directions and exhibiting symmetric photonic bandgaps in every orientation [27]. When a single CLC droplet/microcapsule possesses a perfect radial arrangement of helical axes, the incident light is always nearly parallel to the local helical axis, thereby significantly reducing its sensitivity to the incident angle compared with traditional 1D/2D planar structures. Even in cases where the radial arrangement is not perfectly uniform, integrating such 3D-confined CLC droplets or microcapsules into an array can still enhance angular robustness via ensemble averaging. Since each building block already exhibits low angular sensitivity, Bragg’s law ensures that for any incident light, there always exists a local helical axis aligned with it, effectively averaging out the influence of the incident angle θ. As a result, the angular dependence of the selective reflection’s central wavelength is markedly reduced, yielding structural colors with greatly improved stability and uniformity across viewing angles. This mechanism manifests in a distinctive “Maltese cross” pattern under polarized microscopy, confirming the radial structure. In reflection mode, a constant-color bright spot at the microsphere’s center persists under rotation, demonstrating the significant reduction in angular sensitivity. The reduction of angular dependence in CLC has been widely studied. For instance, Bisoyi et al. [28] achieved omnidirectional lasing from a CLC microshell, where the 3D bandgap with reduced angular sensitivity enables tunable micro lasers. Additionally, Alberto Belmonte et al. [29] fabricated macroscopically suppressed angular sensitivity reflective films by patterning spherically confined CLC particles, yielding large-area coatings with uniform color.

Despite the theoretical advantages of 3D spherical CLCs, micro-scale structural imperfections and geometric factors during measurement can still introduce angle-sensitive color variations in practical applications, thereby affecting experimental repeatability. To ensure data consistency, the measurement procedure must be strictly standardized during optical characterization. The optical readout for CLC sensors in this work primarily adopts a combined spectral-imaging configuration: fiber-coupled spectrometers with optical microscopes for spectral readout, and polarized/non-polarized microscopes for imaging readout, all under fixed collection geometry with near-normal incidence; single droplet readout is used for structural characterization, while ensemble readout is employed for quantitative sensing to improve signal robustness. Typical signal readout systems for CLCs integrate a fiber-coupled spectrometer with an optical microscope, generally employing a fixed-angle illumination (e.g., 90°) and near-normal collection mode to acquire reflection spectra. This geometric setup relies on the assumption of a collimated optical path; however, while the positions of the light source and detector are fixed, the spatial orientation of individual microspheres within the sample relative to the optical axis may exhibit random deviations. Therefore, in addition to standardizing the measurement geometry, optimizing sample preparation is crucial. Signal fluctuations caused by random orientations can be effectively suppressed by encapsulating microspheres in a pre-aligned matrix, solidifying them, or fixing the orientation of droplet arrays using external fields or surfactants. Compared to planar or fiber-based designs, such optimized spherical CLC systems provide more consistent optical performance, making them ideal for high-reliability sensing applications.

### 2.3. Sensing Mechanisms of CLCs

**Figure 3:**
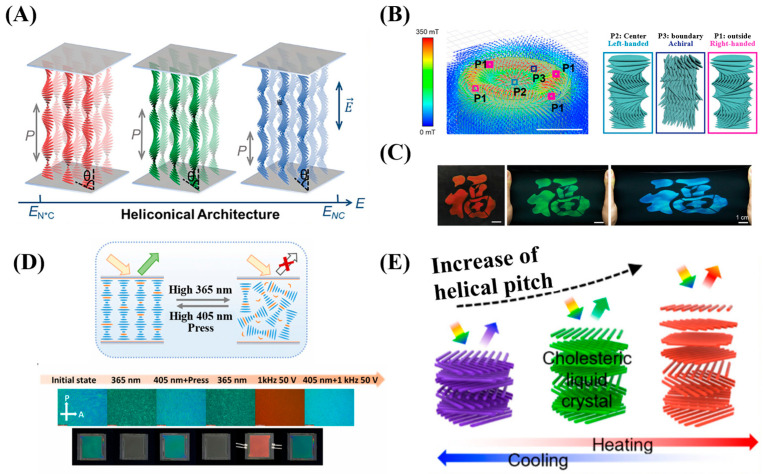
CLC responses to stimuli: (**A**) electric field. E_N*C_ denotes the critical electric field of the chiral nematic phase; the asterisk indicates chirality [31]; (**B**) magnetic field [34]; (**C**) mechanical stress [14]; (**D**) light [35]; (**E**) temperature [13]. Adapted with permission from Ref. [31]. Copyright 2025 John Wiley & Sons—Books. Adapted with permission from Ref. [14]. Copyright 2025 John Wiley & Sons—Books. Adapted with permission from Ref. [35]. Copyright 2024 Royal Society of Chemistry.

The optical properties of CLCs make them highly sensitive to changes in helical pitch induced by external stimuli such as temperature [13,30], chemical environment [18], electric fields [31,32], mechanical stress [14] and light [33], as shown in Figure 3. This sensitivity enables a direct optical readout via variations in color or reflection spectrum, forming a crucial foundation for their applications in sensing. Their thermochromism serves as a prominent example: a shift in color originates from temperature-induced modulation of the helical pitch, which can occur through mechanisms such as phase transitions, changes in the helical twisting power of chiral dopants, and phase separation [23]. Similarly, the pitch can be modulated by other forms of external stimuli that alter the system’s structural parameters: an applied electric or magnetic field reorients the director; mechanical stress directly deforms the helix through compression or extension; and introduced chemical agents (e.g., solvents or analytes) can shift the local order parameter or disrupt the chiral balance of the liquid crystal system. External stimuli can induce transitions between those textures, producing pronounced optical contrast. In 3D spherical confinement (e.g., within droplets), the equilibrium configuration results from the interplay among elastic, chiral, and interfacial anchoring energies [25,26]. Under strong planar anchoring, the molecules align parallel to the droplet surface, and the helical axis tends to distribute radially, forming a radial spherical configuration. In contrast, under strong homeotropic anchoring, the molecules align perpendicular to the surface, and the helical axis tends to orient tangentially, resulting in a fingerprint spherical or polydomain structure containing complex defects [26], which macroscopically exhibits a strongly scattering state. By altering the interfacial anchoring energy or the material’s elastic constants, external stimuli (e.g., chemical adsorption, temperature changes) can drive dynamic transitions between different equilibrium configurations. This topological reconfiguration is often accompanied by abrupt changes in optical signals, such as transparency or scattering intensity, demonstrating high sensitivity to surface interactions. Consequently, such systems are highly suitable for high-sensitivity biomolecular detection and interfacial process monitoring.

Based on the above, the sensing mechanisms of CLCs are categorized into two types:

(1) Bulk-Driven Pitch Modulation Sensing: External stimuli directly alter the intrinsic helical pitch, thereby shifting the Bragg reflection wavelength and enabling color-based sensing.
(2) Interface-Driven Configuration Transition Sensing: Stimuli that alter interfacial conditions or the elastic properties of the system can induce transitions in the microscopic texture or the overall topological configuration of droplets, enabling sensing based on changes in optical patterns or transparency.

## 3. Engineering CLC Nanostructures via Spatial Confinement

CLCs exhibit great potential in sensing applications due to their unique photonic bandgap and stimulus-responsive properties, but they face inherent challenges in practical implementation. Being a soft matter, the fluid nature of CLCs makes it difficult to maintain structural stability over the long term in open environments, and they are susceptible to environmental contamination. More critically, the optical response characteristics of CLCs are highly dependent on their confinement dimensionality and geometry. Low-dimensional confinement often leads to angular sensitivity in signal readout, resulting in measurement errors. Therefore, developing stable, highly sensitive, and user-friendly CLC sensors requires effective spatial confinement strategies.

When LCs are confined to regions where the confinement size is comparable to or smaller than their characteristic lengths, boundary effects decisively influence molecular alignment, defect formation, and overall physical response. Such systems are termed “confined liquid crystal systems” [25,36]. More specifically in this context, n-dimensional confinement refers to a situation where a system is confined along n independent spatial dimensions, such that the confinement length scales are comparable to or smaller than one of its characteristic lengths (e.g., the helical pitch). Accordingly, confined CLCs encompass several forms across dimensions: 1D (e.g., cells, thin films), 2D (e.g., fibers, microchannels), and 3D (e.g., droplets, microcapsules), among others. Confined liquid crystal systems are not only widespread in biological organisms (such as the lipid bilayers in cell membranes [37] and the structural-color layers in certain beetle shells [38]) but are also extensively studied as an important paradigm for material design. For instance, confining LCs to spherical interfaces can induce the formation of ordered structures with topological defects. Such functional colloidal particles can serve as mesoscale “building blocks” for the self-assembly of photonic crystals or responsive colloidal arrays [39,40]. Owing to their typically higher specific surface area and restricted volume, interfacial effects are greatly amplified in confined systems, leading to significantly enhanced sensitivity to external fields or chemical stimuli. This creates new opportunities for developing high-performance optoelectronic and sensing devices. Therefore, this section systematically explores, from an engineering perspective, strategies for the nanostructural regulation of CLCs through traditional 1D/2D strategies and emerging microfluidic technologies. It will focus on analyzing the revolutionary advantages of microfluidic techniques in overcoming the limitations of conventional methods.

### 3.1. Conventional Strategies for 1D and 2D Confinement of CLCs

Before microfluidic technology became prevalent, conventional physical or chemical methods were utilized to confine CLCs in sensor device construction. These strategies were chiefly confined within 1D and 2D geometric spaces.

Within 1D confinement, the prevailing method is the “sandwich” liquid crystal cell. In this approach, the CLC is confined between two parallel glass substrates. The inner surfaces of the substrates are coated with an alignment layer, such as polyimide (PI), and then rubbed to induce planar alignment of the CLC molecules. For thin film, conventional fabrication techniques like spin-coating [41], blade-coating [42], inkjet printing [43,44] and in situ polymerization [45] are employed. These traditional methods benefit from well-established processes and readily available equipment, contributing significantly to the development of early sensor devices. Within 2D confinement, a common approach is to encapsulate CLCs within capillaries or coat them onto cylindrical fibers. Fiber preparation methods such as electrospinning [46,47], wet spinning [48], and melt spinning [49] are employed. These traditional confinement structures have allowed researchers to create diverse sensing applications based on low-dimensionally confined CLCs.

**Figure 4:**
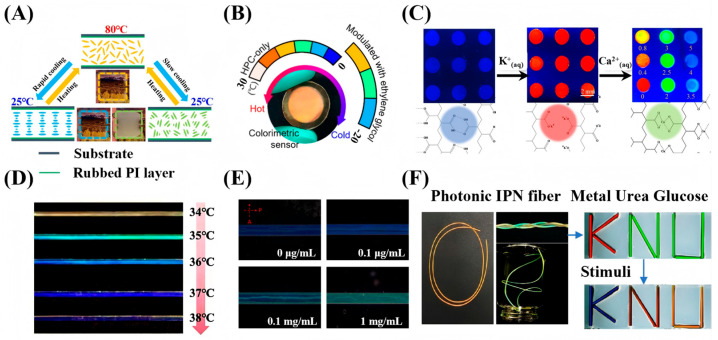
Sensing applications based on low-dimensional confined CLCs. (**A**) Thin-film cell for temperature sensing [51]. (**B**) Flexible CLC film encapsulated in PDMS for temperature sensing [13]. (**C**) Photonic interpenetrating IPN film array for calcium ion detection [53]. (**D**) Thermoresponsive CLC elastomer fiber [55]. (**E**) BSA sensing using microchannel-confined CLCs [57]. (**F**) Photonic IPN fiber array for the detection of urea and glucose [58]. Adapted with permission from Ref. [51]. Copyright 2023 American Chemical Society. Adapted with permission from Ref. [53]. Copyright 2019 Elsevier. Adapted with permission from Ref. [55]. Copyright 2024 Elsevier. Adapted with permission from Ref. [58]. Copyright 2024 American Chemical Society.

In 1D-confined CLCs sensing, the device design has gradually evolved from hard liquid crystal cells and flexible films to CLC-hydrogel interpenetrating network films, demonstrating diverse sensing mechanisms and performance improvements. For instance, Spengler et al. [50] developed a photonic sensor for detecting NO_2_ gas by spin-coating a CLC photonic film on a polyvinyl alcohol (PVA) alignment layer. The sensor exhibited high selectivity in a 400 ppm NO_2_ atmosphere, with a detection limit of 100 ppm. In another study, Yin et al. [51] utilized polymerizable CLCs encapsulated in parallel-aligned liquid crystal cells (20 μm in thickness). By controlling the cooling rate, they achieved a controllable transformation of the CLC texture between the planar and the focal conic states, thereby constructing a sensing platform for the visual detection of temperature, as shown in Figure 4A. These two studies exemplify two typical sensing mechanisms based on pitch modulation and texture transformation, respectively. In the field of polymer film sensors, Mu et al. [52] constructed sensors for cholesterol and malathion detection based on a composite system of CLC elastomers and polyelectrolytes, achieving detection limits of 0.08 mM and 0.18 ng/mL, respectively. As shown in Figure 4B, Yi et al. [13] developed a low-temperature sensing film by incorporating ethylene glycol into LCs. Encapsulated as a thin film using a flexible microcavity structure, the sensor enables continuous visual temperature monitoring over a range of −20 °C to 25 °C. Building upon the foundation of interpenetrating network systems, Myung et al. [53] prepared photonic interpenetrating polymer network (IPN) array films by interpenetrating reactive CLCs with polyacrylic acid (PAA) networks. Based on this structure, they developed a multifunctional sensor capable of detecting humidity and calcium ions (Ca^2+^), achieving a detection limit of 0.35 mM and a linear range of 0.4–3.5 mM for Ca^2+^. This sensor has been successfully applied to the detection of calcium ions in human serum and saliva, as shown in Figure 4C. In related work, Yeh et al. [54] fabricated IPN lattice devices with a feature size of ~8 μm via a capillary filling method using a functionalized PAA hydrogel. These devices were used to sense methanol, ethanol, and their mixtures, with detection limits of 1.67% for pure ethanol and 1.32% for pure methanol.

Common implementations of 2D-confined CLC sensing include CLC elastomer fibers, microchannel-confined CLCs, and photonic IPN fibers. In the first category, Fu et al. [55] fabricated PDMS/CLC core-sheath fibers (approximately 539 μm in diameter) via dry spinning for temperature sensing. Sensors based on these fibers reliably measured human body temperature between 34 °C and 38 °C while demonstrating good sweat resistance and stability, as shown in Figure 4D. Similarly, Zhao et al. [56] obtained solid elastic fibers approximately 1 mm in diameter using coaxial dry spinning, which enabled visual temperature warning and indication. Turning to microchannel confinement, Wang et al. [57] utilized microfluidic channels to achieve bidirectional confinement of CLCs, thereby creating a biosensing chip for highly sensitive detection of bovine serum albumin (BSA) and its immune complexes. This chip exhibited a linear detection range from 0.1 μg/mL to 1.0 mg/mL and a limit of detection (LOD) around 1.0 ng/mL, as shown in Figure 4E. Finally, in the approach using IPN fibers, Adane et al. [58] produced fibers (~600 μm in diameter) by injecting reactive liquid crystal mixtures into PTFE tubes followed by UV curing. This platform enabled a multifunctional sensor capable of simultaneously detecting humidity, Ca^2+^, urea, and glucose, with respective detection limits of 7.86% relative humidity, 0.07 mM, 2.54 mM, and 0.76 mM, respectively, as shown in Figure 4F. The sensor also showed good selectivity and cyclic stability.

These examples show that diverse structural designs enable practical, stable sensors via 1D/2D confinement. However, while such traditional methods laid the foundation for CLC sensors, they still suffer from fundamental limitations in achieving high-performance sensing. First, dimensional limitations lead to stability issues. Due to the inherent fluidity of CLCs, molecules in 1D fibers or 2D films are susceptible to external perturbations that cause flow, resulting in texture disruption or signal drift. Second, and most critically, there is a pronounced angular sensitivity. As dictated by Bragg’s law, the structural color wavelength reflected by CLCs is highly dependent on the angle (θ) between the incident light and the helical axis. Under 1D/2D confinement, if the helical axis is not aligned with the viewing direction, the reflected color exhibits strong angular sensitivity. Consequently, the optical response can vary dramatically with viewing angle, and the reflection may even disappear. This leads to significant reading errors under real-world, non-ideal observation conditions. Moreover, traditional methods are limited in fabricating complex 3D structures with high dimensional precision. Attaining micro- or nanoscale accuracy in parameters like film thickness or fiber diameter remains difficult with conventional approaches. The fabrication of microspheres featuring complex internal hierarchies (e.g., core–shell or Janus structures) is even more challenging. These limitations restrict the potential for enhancing sensor multifunctionality and response speed. Consequently, developing a new technology that enables true 3D spatial confinement, mitigates angular sensitivity, and allows precise microstructural control has become critically important.

### 3.2. Microfluidic Strategies for 3D Confinement of CLCs

**Figure 5:**
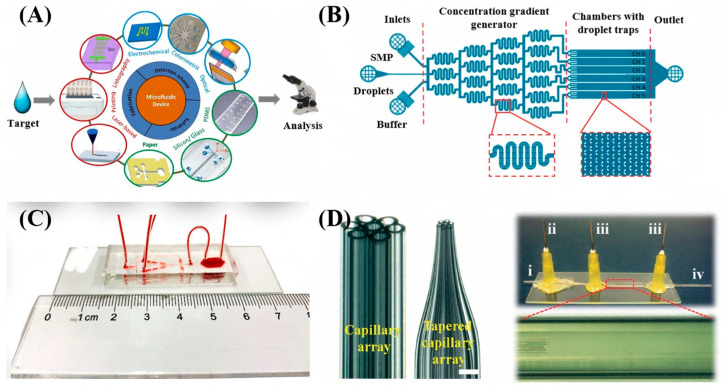
Schematics of microfluidic device structures and their applications: (**A**) Microfluidic device for sensing and analysis [75]. (**B**) Microfluidics-assisted liquid crystal generation and on-chip confinement [76]. (**C**) Photograph of a PDMS chip [77]. (**D**) Right panel: photographs of the seven-hole annular capillary array before and after thinning (scale bar: 1 mm). Left panel: micrograph of the assembled capillary microfluidic device, where i–iv represent the inner channel, middle channel, outer channel, and collection channel, respectively [78]. Adapted with permission from Ref. [75]. Copyright 2022 Elsevier. Adapted with permission from Ref. [78]. Copyright 2014 Royal Society of Chemistry.

To overcome the limitations of traditional 1D/2D constraints, microfluidic technology, as shown in Figure 5A which is a revolutionary tool capable of precisely manipulating minute fluid volumes, has been introduced into the 3D spatial confinement of CLCs [59,60]. The development of microfluidic chips has evolved through three key phases. It began in 1990 with the introduction of the micro-total analysis system (μ-TAS) concept and early demonstrations of chip electrophoresis by A. Manz et al. [61]. In the subsequent phase, chip electrophoresis gained wider adoption, leading to advances in integrated chip design. A notable milestone was achieved by the Unger et al., which utilized multilayer soft lithography to create pneumatic micropumps and valves, culminating in a highly integrated chip featuring thousands of valves and hundreds of reactors, published in *Science* in 2002 [62,63]. In recent years, understanding and innovation in microfluidics have accelerated, yielding a continuous stream of new technologies and methods. The field has expanded well beyond chemistry into biology, physics, and medicine. Among various microfluidic technologies, droplet microfluidics demonstrates unique advantages. It focuses on generating droplets using immiscible phases, effectively dividing the fluid into independently controllable micro-units. Compared to continuous-flow systems, droplet microfluidics not only significantly reduces reagent consumption but also offers greater throughput and better scalability [60,64].

Optofluidics integrates optics and microfluidics at the microscale, enabling the manipulation of light using fluids or the control of fluids and particles via light [65,66,67]. This convergence offers enhanced precision and novel functionalities. A prime example within this field is LC microfluidics, which represents the integration of LC properties with microfluidic control, further advancing these capabilities [68,69,70,71]. Research has a dual focus: (1) using microfluidic confinement to create self-assembled LC structures for on-chip sensing platforms; and (2) utilizing LC’s stimuli-responsiveness as smart materials to microfluidic systems. As shown in Figure 5B, this microfluidic chip generates confined CLC droplets and incorporates sensors on its substrate. A notable example is the “lab-on-a-display” concept introduced in 2006, which uses liquid crystal displays to generate patterned electric fields for non-contact particle manipulation. This strategy has enabled extensive and diverse applications [72,73,74].

#### 3.2.1. Design and Fabrication of Microfluidic Devices for CLC Emulsions

**Figure 6:**
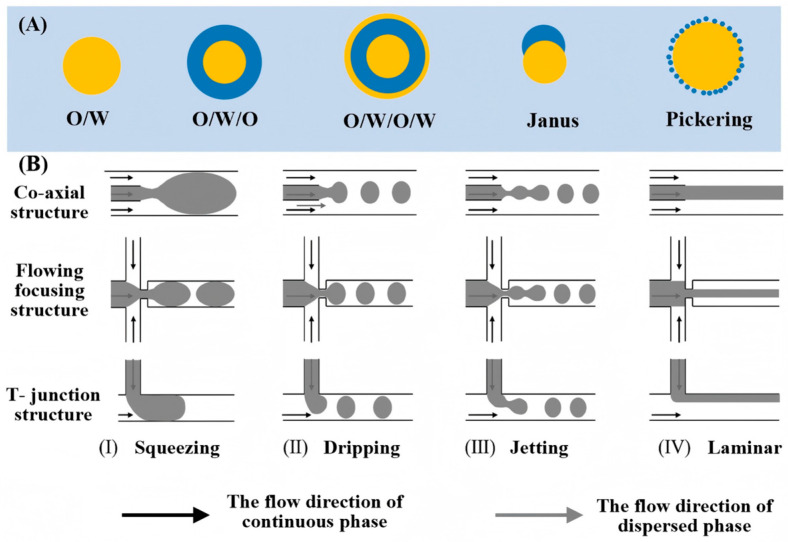
Schematic diagrams of microfluidic-based emulsion generation. (**A**) Microstructural schematics of different types of emulsions. (**B**) Typical microfluidic chip geometries for emulsion generation [81]. Adapted with permission from Ref. [81]. Copyright 2024 John Wiley and Sons.

An emulsion is a dispersion of one liquid as droplets within another, immiscible liquid. Simple emulsions feature droplets directly suspended in a continuous phase. More complex architectures include multilayer emulsions, where droplets encapsulate smaller, immiscible droplets, creating nested layers. Systems with three immiscible phases can form Janus emulsion droplets. Alternatively, in the absence of surfactants, colloidal nanoparticles can adsorb at the interface to stabilize Pickering emulsions. A fundamental example of a 3D confined soft material is the CLC emulsion droplet, typically formed as a single oil-in-water (O/W) structure [79,80]. The common types of emulsions are shown in Figure 6A.

The controlled generation of such emulsions, especially with complex internal order like CLCs, heavily relies on microfluidic technology. The fabrication of these devices requires careful selection of compatible materials and optimized channel geometries. Common substrate materials include inorganic options, polymers, and paper-based substrates [82], as shown in Figure 5C,D. Polydimethylsiloxane (PDMS) is extensively used due to its ease of molding via soft-lithography, transparency, and flexibility [83]. Its native hydrophobic surface facilitates water-in-oil (W/O) droplet generation, though hydrophilic treatment is necessary for aqueous-continuous phases or multiple emulsions. A significant limitation of PDMS is its swelling in strong organic solvents, reducing stability for complex emulsion formulations [84]. Capillary-based microfluidic devices, pioneered by Utada et al. [85], offer an alternative by using aligned glass capillaries. This platform provides superior chemical resistance, easier surface modification, and enables the production of highly precise single and multiple emulsions with tunable sizes. However, manual assembly can lead to reproducibility challenges. Other materials like paper [86] and 3D-printed substrates [87,88] are also explored, each with specific trade-offs.

The geometry of microfluidic channels is as crucial as material selection, directly governing droplet formation. As shown in Figure 6B, key designs include T-junction, co-flow, and flow-focusing structures [82,89]. The T-junction, pioneered by Thorsen et al. [90], intersects dispersed and continuous phases at an angle, using shear and pressure to form droplets via head extension and neck thinning. It is simple to fabricate, requires no precise alignment, and produces highly monodisperse droplets (CV < 2%) [91], making it ideal for single emulsions. The co-flow configuration, introduced by Umbanhowa et al. [92], aligns phases coaxially for parallel flow, generating droplets in a dripping regime with low polydispersity (CV < 3%), though droplet sizes are typically large (hundreds of micrometers). In contrast, flow-focusing forces the dispersed phase through a narrow orifice via symmetric squeezing from two opposing continuous streams [93]. This amplifies shear, breaking the jet into highly uniform droplets as small as several hundred nanometers, enabling much finer control than basic co-flow designs.

**Figure 7:**
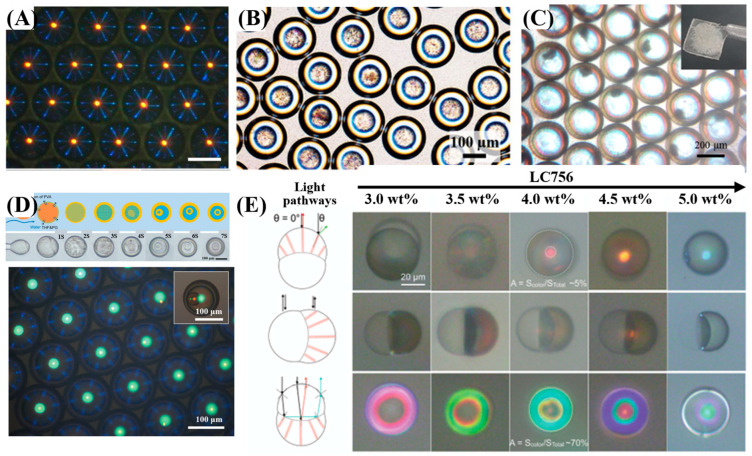
Microfluidic-assisted fabrication of 3D-confined CLCs: (**A**) Droplet [96], (**B**) Microcapsule (double emulsion) [97], (**C**) Shell [98], (**D**) Onion-like structure (triple emulsion) [99], (**E**) Janus structure [100]. Adapted with permission from Ref. [96]. Copyright 2021 Elsevier. Adapted with permission from Ref. [97]. Copyright 2022 John Wiley & Sons—Books. Adapted with permission from Ref. [98]. Copyright 2019 American Chemical Society. Adapted with permission from Ref. [99]. Copyright 2020 John Wiley & Sons—Books. Adapted with permission from Ref. [100]. Copyright 2023 John Wiley & Sons—Books.

Currently, microfluidic devices for confined CLCs are primarily based on PDMS and glass capillary chips, with the flow-focusing geometry being the most commonly used [20]. For preparing 3D-confined CLC emulsions, surface wettability of the microfluidic device is paramount. PDMS or glass capillary chips require hydrophilic or hydrophobic treatment accordingly [94,95]. PDMS is often treated with oxygen plasma or PVA coating. Glass capillaries can be chemically modified using silane coupling agents—for example, 2-[methoxy(polyethyleneoxy)propyl] trimethoxysilane (mPEG-Silane) for hydrophilicity, or octyltrimethoxysilane (OTMS) for hydrophobicity. While PDMS chips offer convenience, glass capillary devices are more prevalent for generating double or triple CLC emulsions due to their stability and modifiability. Using glass capillary devices, single CLC emulsion droplets are formed in an O/W system with CLCs as the oil phase. By employing coaxial multi-channel devices, more complex architectures are achievable: W/O/W systems yield double emulsions with a CLC shell, while O/W/O systems produce double emulsions with a CLC core. Further stacking of coaxial capillaries enables even greater complexity, such as triple-layered or multilayer liquid crystal microspheres, using multiphase systems like O/W/O/W. Consequently, they have been widely adopted for the controlled fabrication of various CLC structures, including those shown in Figure 7: (A) droplets [96], (B) microcapsules [97], (C) shells [98], (D) onion-like structures [99], and (E) Janus structures [100].

In microfluidic CLC droplet formation, surfactants both stabilize the interface and control LC anchoring through their amphiphilic nature. Surfactant HLB determines interface type: >7 stabilizes O/W, lower HLB stabilizes W/O [79,80]. Choice of surfactant or channel modification directs planar or perpendicular anchoring, governing whether helices align radially or tangentially in the droplet. For instance, planar anchoring readily occurs in PVA solution, while surfactants such as sodium dodecyl sulfate (SDS) or cetyltrimethylammonium bromide (CTAB) tend to induce perpendicular anchoring [101]. This control over anchoring directly governs the microscopic configuration and optical response of confined LC droplets. In NLC droplets, common configurations include bipolar (with defects at the poles) or radial (with a central defect) alignment [102]. In CLC, configurations are governed by pitch, droplet size, chiral strength, and surface anchoring [26,103]. When adsorbed amphiphilic molecules switch the interfacial alignment from planar to homeotropic, a flashlight-like pattern emerges, accompanied by significant textural and optical changes [104].

Therefore, to achieve the microfluidic fabrication of 3D confined CLC systems, the following aspects must be considered together: device design and geometry, surface wettability modification, multi-channel device integration, and surfactant selection.

#### 3.2.2. Formation Mechanisms and Guidelines for CLC Emulsions in Microfluidic Devices

**Figure 8:**
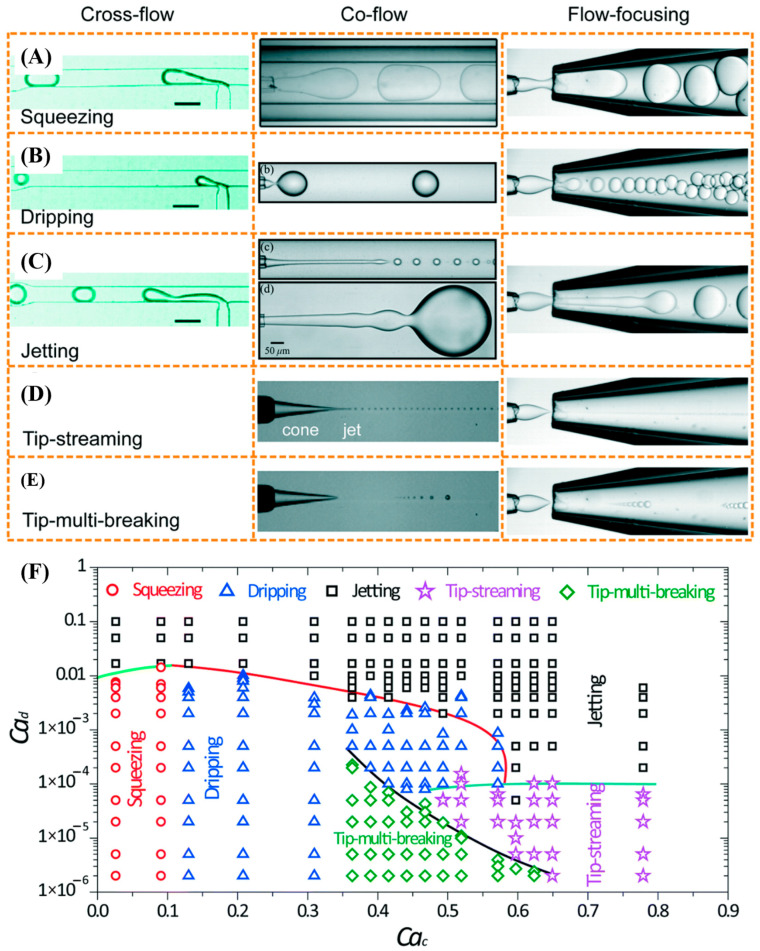
Images of droplet generation with different modes in cross-flow, co-flow and microcapillary flow-focusing geometries. (**A**) Squeezing mode [105]. (**B**) Dripping mode [105]. (**C**) Jetting mode [105]. (**D**) Tip-streaming mode [105]. (**E**) Tip-multi-breaking mode [105]. (**F**) Phase diagram in (Ca_c_, Ca_d_) plane for various modes observed in microcapillary flow-focusing devices [105]. Adapted with permission from Ref. [105]. Copyright 2017 Royal Society of Chemistry.

In microfluidic devices, CLC emulsion droplets form when applied forces overcome the interfacial tension between the continuous phase and the CLC (dispersed) phase, leading to flow instability and subsequent droplet breakup. This process can be precisely controlled, even in fixed geometries, by adjusting fluid properties such as viscosity and interfacial tension, which are particularly important given the anisotropic nature and viscoelasticity of CLCs. At this scale, droplet breakup is governed by dimensionless numbers such as the Reynolds number (Re), Capillary number (Ca), and Weber number (We), which relate to fluid properties, channel geometry, and flow conditions [91,105]. Their values and ratios directly characterize the underlying physics of the droplet formation process. The capillary number (Ca), representing the ratio of viscous forces to interfacial tension, governs droplet formation in low-Reynolds-number laminar flow. As Ca increases, the dominant mechanism transitions sequentially through three stages: squeezing (Ca < 10^−2^), dripping (Ca > 10^−2^), and jetting (at higher Ca), as shown in Figure 8. The Weber number (We) quantifies the ratio of inertial forces to interfacial tension and predominates in microfluidic systems where inertial effects are significant. At high We, inertia overcomes interfacial tension, leading to droplet breakup and the formation of smaller droplets, though often at the cost of increased flow instability and droplet polydispersity. Collectively, these dimensionless numbers form the fundamental framework for guiding the generation of CLC emulsions in microfluidic devices, as summarized in Table 1.

#### 3.2.3. Structural Design and Performance in 3D-Confined LC Sensing Chip

In the field of microfluidic sensing, NLCs have been extensively studied [69,106]. NLCs rely on refractive index anisotropy, requiring polarized light for observation and typically showing intensity-based signals. In contrast, CLCs have a helical structure that enables selective reflection and a tunable photonic bandgap. Their pitch is highly sensitive to external stimuli, allowing detection through direct color/wavelength shifts without complex optics. CLCs also provide multidimensional outputs (color, texture, transmittance) and respond to various parameters, making them ideal for rapid, visual sensing [20,101,107].

The structural design of CLCs emulsion sensing units directly influences their response traits and overall performance. Single emulsions, the simplest form, allow target substances adsorbed at the interface to alter interfacial energy and trigger CLCs reorientation, producing distinct optical signals. They offer sensitive response and clear signals but suffer from limited long-term stability. In contrast, O/W/O double emulsions (microcapsules) encapsulate the CLCs within a core, improving protection at the cost of slower analyte diffusion, resulting in delayed response and reduced signal intensity. Furthermore, multiple emulsions enhance encapsulation and isolation by increasing the number of layers, enabling multistage programmable responses. However, they involve the most complex preparation, pose challenges in stability control, and significantly impact mass transfer and response kinetics. In another shell structure, CLCs form a continuous shell around an inner aqueous phase, creating a dual interface that enables dual-interface response. However, this configuration is very unstable. The CLC shell tends to rupture or coalesce, and the resulting optical pattern is challenging to interpret. In contrast, the Janus structure combines a CLC sensing half with a functional half (e.g., for magnetic control or probe attachment) into a single emulsion. This enables directional sensing, movement, and target collection, though the emulsion is more complex to produce. It should be noted that if the CLC in shell or Janus structures, or the microcapsules themselves, lack perfect spherical symmetry (e.g., due to uneven pressure or osmotic effects), their optical response will still exhibit angular dependence. Each structural type therefore involves trade-offs in sensitivity, stability, response speed, and functional integration, and should be selected based on specific sensing requirements.

Droplet size and shape are critical for tuning sensing performance. Smaller droplets offer a larger surface area, which accelerates target diffusion and shortens response time (RT). However, excessively small droplets can yield weaker signals due to limited volume, and are prone to evaporation, leading to concentration shifts and baseline drift. Stability often requires controlled environments or oil-sealing. Uniform droplet size, enabled by microfluidics, ensures repeatable measurements by maintaining consistent optical paths. Additionally, droplet curvature influences light propagation; highly curved droplets can act as optical resonators, enhancing light–matter interaction and sensitivity. In summary, system design requires balancing response speed, signal strength, stability, and optical sensitivity.

Downstream droplet manipulation in microfluidics, which encompasses splitting, merging, sorting, transport, and positioning, serves as a critical link between droplet generation and subsequent detection, analysis, and synthesis. This technical frame-work has been extensively studied and developed [89,108,109]. Building on these capabilities, the microfluidic platform offers a unique approach for precise spatial confinement and functionalization of liquid crystals, thereby advancing the development of high-performance, compact CLC sensors. However, real-time observation of 3D-confined CLC in solution is challenging due to their high fluidity. One approach involves inkjet printing droplets onto a solid substrate for integration into microfluidic channels, though such structures sacrifice some 3D symmetry and are prone to detachment under flow shear [110]. Alternatively, microstructures such as micropillars [111], dovetail structures [76], small grooves [97,107], etc., can be designed to trap and isolate droplets into arrays within independent chambers, enabling stable confinement in flowing environments. In addition, to achieve reliable on-chip CLC sensing, apart from precisely fabricating the structure, developing compatible downstream droplet manipulation techniques (such as selective sampling, quantitative merging, arrayed docking, etc.) is crucial for achieving repeatable generation, internal calibration, multiple tests and standardized measurements. In contrast to 3D confinement, a more direct strategy utilizes microfluidic technology to construct two fundamental types of sensing interfaces: LC-solid and LC-aqueous [101,112]. However, in such structures, CLCs are not in a 3D-confined state and therefore do not constitute the primary focus of this study.

## 4. Sensing Applications of 3D Confined CLCs

Based on the microfluidic engineered CLC 3D-structure discussed in Section 3, this section focuses on their specific applications in physical, chemical, and biosensing. Specific cases are presented to illustrate how their unique optical properties enable highly sensitive and visualized detection of various target stimuli.

### 4.1. Physical Sensing

**Figure 9:**
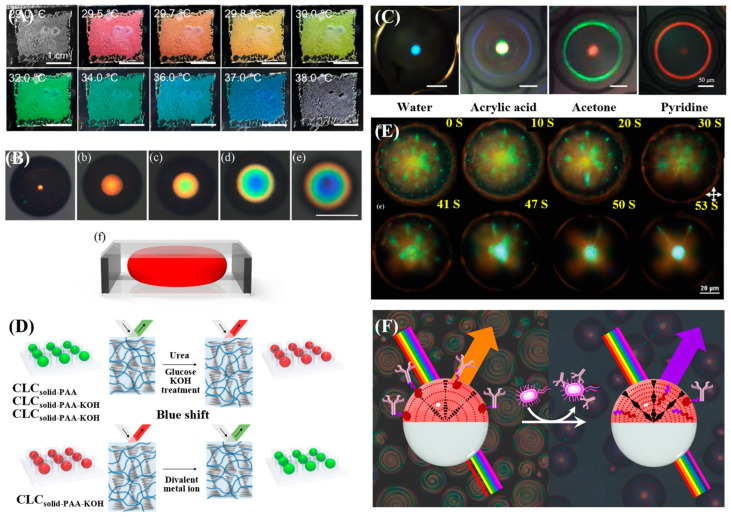
3D-confined CLC sensors for various applications: (**A**) Temperature sensing using a droplet [12]. (**B**) Stress sensing with a particle [96]. (**C**) Solvent detection based on a shell structure [98]; (**D**) Particle-based sensing of pH, divalent metal ions (Ca^2+^, Mg^2+^), urea, and glucose [107]. (**E**) Droplet sensor for DCA detection [114]. (**F**) Janus structure for Salmonella detection [115]. Adapted with permission from Ref. [12]. Copyright 2025 Elsevier. Adapted with permission from Ref. [96]. Copyright 2021 Elsevier. Adapted with permission from Ref. [98]. Copyright 2019 American Chemical Society. Adapted with permission from Ref. [107]. Copyright 2020 American Chemical Society. Adapted with permission from Ref. [114]. Copyright 2021 Royal Society of Chemistry. Adapted with permission from Ref. [115]. Copyright 2021 American Chemical Society.

Among these applications, temperature sensing is one of the most well-established. It relies on the thermochromic response of CLCs, in which a temperature change alters the helical pitch, causing a shift in the reflected wavelength. For example, Oh et al. [12] fabricated CLC droplets approximately 100 μm in diameter via microfluidics, encapsulating them within a polymer matrix to construct a thermochromic sensor. With exceptionally high sensitivity (Δλ/ΔT > 100 nm/°C), the sensor yields a remarkable 174 nm wavelength shift over the range of 26.8–27.8 °C, as shown in Figure 9A. Besides directly embedding CLC droplets into a matrix, another common strategy is to confine the CLCs within a microcapsule structure, which allows further tuning of its thermal response. For instance, Pan et al. [113] used a glass capillary device to produce O/W/O double-emulsion droplets, which served as templates for preparing monodisperse CLC microcapsules with a core diameter constrained to 105–113 μm. These capsules demonstrated thermochromic behavior, showing distinct responses in two specific temperature ranges: 52–56 °C and 33–38 °C. Kim et al. [97] further prepared CLC microcapsules with the aim of broadening the temperature measurement range. Adjusting the ratios of three cholesteric esters enabled precise regulation of the response range from 2 to 37 °C, accompanied by a full visible-spectrum color shift. A detection limit of 0.05 °C (spectral resolution: 5 nm) was achieved, making them promising for high-precision local temperature monitoring.

In addition to sensing temperature, pressure detection is a widely used application for physical sensors. Such 3D-confined CLCs are particularly advantageous, as their reflected light shifts in response to stress-induced changes in their helical axis orientation or pitch. Whereas earlier designs were mostly limited to low-dimensional structures such as fibers and films, microfluidic techniques now allow the fabrication of highly sensitive 3D elastic microspheres or complex arrays. In 2021, Lim et al. [96] fabricated solid photonic CLC-IPN spheres with a confinement scale of 80 μm. As shown in Figure 9B, under compressive strain, the reflection color of these spheres shifts from orange to blue as the radius compression ratio increases from 0 to 0.67. Likewise, Xie et al. [15] further extended the confinement scale to a system of CLC droplets ranging from 5 to 75 μm in diameter. Their resulting sensor can detect curvature radii within the range of 5.5 to 22.5 mm, corresponding to separation distances of 10–50 μm. Together, these works demonstrate that CLC-based materials can convert small deformations into clear optical signals for visual force feedback, offering great potential in soft robotics and wearables.

### 4.2. Chemical Sensing

For chemical sensing applications, 3D-confined CLCs can serve as sensors for volatile organic compounds (VOCs), pH, H_2_O, and ions. VOC detection is primarily achieved through solvent-induced refractive index changes or structural swelling. For example, Shang et al. [116] prepared CLC particles (92–149 μm) via microfluidics to develop sensors for detecting various organic solvents (e.g., ethanol, methanol). These sensors can both qualitatively distinguish between different solvent types and quantitatively determine the tetrahydrofuran (THF)content in THF–water mixtures. In addition to particle morphology, microfluidic technology can also be used to prepare solid CLC shell structures. For instance, the solid CLC shells developed by Myung et al. [98] show distinct swelling behaviors in different solvents, as shown in Figure 9C. Such shell structures allow precise control over shell thickness and permeability through microfluidics, thereby optimizing the mass transfer rate of solvent molecules and significantly improving detection selectivity and response speed.

pH and ion sensing are often achieved by introducing functional monomers or hydrogel networks into CLC systems. For instance, Kim et al. [107] developed a solid-state droplet array. Through the functionalization with different monomers, this array enables multiplexed detection of a broader range of targets, including pH, divalent metal ions (e.g., Ca^2+^ and Mg^2+^), urea, and glucose, as shown in Figure 9D. Microfluidic technology provides these droplets with high monodispersity and reproducibility, which are essential for constructing arrayed sensors for simultaneous multi-analyte detection. By modifying different functional groups at the droplet interface, 3D-confined CLC sensors with high selectivity for specific ions can be designed.

### 4.3. Biosensing

Biosensing represents one of the most promising applications for 3D-confined CLCs. It primarily leverages specific biomolecular interactions at the CLC interface to either alter the helical pitch (leading to a color shift) or modify the surface anchoring (causing a structural reconfiguration), both of which result in a detectable change in optical signals. The detection of biomolecules primarily relies on mechanisms such as competitive surfactant adsorption or specific ligand–receptor binding. As shown in Figure 9E, Gollapelli et al. [114] developed stable CLC droplets composed of PVA and sodium lauryl sulfate (SC12S). Their sensing mechanism relies on surfactant competitive adsorption, which triggers an anchoring transition inside the droplets. This enables highly sensitive detection of specific biomolecules like cholic acid (CA) and deoxycholic acid (DCA).

Moreover, sensing platforms relying on the volume phase transition of hydrogels have been extensively developed for detecting small biomolecules, including glucose and urea. Microfluidics offers exceptional droplet uniformity and permits precise chemical tuning of the CLC interface, which is critical for achieving highly specific biosensing with a suppressed background. The application of microfluidics has enabled increasingly advanced configurations of CLC structures for pathogen detection. As shown in Figure 9F, Concellón et al. [115] demonstrated this principle by using evaporation-induced phase separation technique to create Janus droplets comprising a CLC phase and a fluorocarbon oil phase. Based on a boronic acid-functionalized polymer surfactant that provides specific, reversible IgG binding, this system successfully detected the foodborne pathogen Salmonella. Compared with droplet or microcapsule, the Janus structure can integrate capture recognition and signal conversion on the two sides of a droplet, or equip each side with different properties, thereby enabling multimodal collaborative functions. Such complex 3D structures produced using microfluidic techniques not only allow for highly sensitive pathogen detection but also serve as the foundation for multifunctional, integrated sensing platforms designed for complex environments.

In summary, the sensing performance of 3D-confined CLC structures fundamentally depends on their confined micro/nano-scale architectures and the tailored responsiveness of their constituent materials. To clarify the relationships between structure, function, and performance, Table 2 summarizes the key characteristics of such sensors, presenting a systematic comparison of their confinement geometries (e.g., droplets, capsules, shells), target analytes, and quantitative sensing indicators. Therefore, this overview both synthesizes the current state of the art and contributes to the ongoing discussion on designing effective CLC-based sensors.

## 5. Conclusions and Outlook

In conclusion, this review has systematically summarized the sensing principles, structural engineering, and applications of CLCs. CLCs exhibit unique selective reflection and structural color due to their periodic helical superstructure and resulting 1D photonic bandgap, forming the basis for optical sensing. Their sensing mechanisms are primarily divided into two categories: direct modulation of the helical pitch by external stimuli for bulk-driven sensing, and changes in interfacial conditions or elasticity that induce microscopic texture or topological transitions for interface-driven sensing. However, as soft materials, CLCs face significant challenges in practical applications, including structural instability, signal drift, and particularly the strong viewing-angle dependence of their optical response. Traditional 1D/2D confinement methods, despite being well-established, struggle to overcome these inherent limitations. Consequently, microfluidic technology has been introduced to achieve precise 3D spatial confinement. By manipulating multiphase flows, this technique enables the controllable fabrication of monodisperse droplets and microcapsules, and further allows the construction of complex topological architectures such as core–shell and Janus structures. By tuning interfacial anchoring conditions, it facilitates the active design of optical responses. 3D confinement significantly reduces angular dependence while enhancing interfacial sensitivity and stability due to the increased specific surface area and restricted volume. Consequently, 3D CLC structures fabricated via microfluidics showcase broad sensing potential. They provide high sensitivity for physical parameters like temperature and stress. In chemical sensing, they allow for the specific detection of VOCs, ions, and small biomolecules. Their biosensing function works through molecular binding at the interface, detecting everything from biomarkers to pathogens. Advanced designs like Janus droplets exemplify the integration of recognition and response functions. In summary, by combining their unique capability to convert molecular-level information into macroscopic optical signals with advanced confinement and fabrication strategies, CLCs have emerged as key candidate materials for driving innovation in biomedical, environmental monitoring, and industrial applications.

Despite these promising capabilities, the performance of CLC-based microfluidic sensors is fundamentally governed by their geometric design, specifically size and curvature. Leveraging microfluidics, we can engineer tailored 3D CLC microstructures like droplets or Janus particles with precise control over these parameters. Their large specific surface area enables rapid analyte diffusion and full utilization of the sensing volume, leading to faster response. While boosting the specific surface area, size reduction can also impair the anchoring and elastic balance of the CLCs. This may lead to defects and textural instability, thereby reducing the reliability and repeatability of optical readouts. High-curvature interfaces enhance local fields and signal amplification but intensify challenges in surface functionalization. These include difficulties in the uniform, high-density immobilization of receptors on curved surfaces, complicated by the need to modify each side of Janus structures differently. Simultaneously, reduced size weakens optical signals, necessitating a balance between detector sensitivity and signal-to-noise ratio in the design. At micro-scale and high curvature, sensors become delicate as small perturbations can induce major reconfigurations and raise noise sensitivity. Therefore, the central challenge is to balance the co-optimization of size and curvature to achieve high sensitivity and speed while ensuring reliable and stable signals.

Signal interpretation complexity presents another key challenge for practical implementation. The output of CLC sensors depends on changes in CLC molecular alignment, often involving not just spectral shifts but complex optical phenomena—such as topological defect motion, texture pattern reorganization, and multi color domain mixing. This multidimensional response makes quantification difficult. The key challenge is converting complex patterns and colors into a single, easy-to-read signal in a consistent way. Moreover, traditional spectral analyzers cannot easily capture such dynamic, spatially coupled information, and current reliance on high resolution microscopy limits portability and impedes point of care use. Thus, creating new detection approaches that balance high information content with easy readout is very essential.

Furthermore, in practical applications, when sensors are exposed to complex samples such as blood or wastewater, their selectivity and anti-interference capabilities are often insufficient to ensure detection accuracy. At the same time, non-specific adsorption readily occurs on the surface of droplets or microcapsules, where impurities disrupt the alignment of LCs, leading to false positive signals. Additionally, the action of analytes is constrained by passive diffusion, resulting in significantly delayed responses.

To address these practical limitations and advance the field, future research on 3D-confined CLC-based sensors should target several key areas. The following directions are proposed to overcome existing challenges and enhance performance:

1. Collaborative Optimization of Size and Curvature: A thorough understanding of how size and curvature interact in 3D-confined CLCs is required. Using both computer simulations and lab experiments to find the best balance: making the droplet surface large enough to capture targets quickly, while keeping its internal structure stable. A clear guide mapping the influence of size and shape on color signal and reliability is essential for designing optimized practical sensors.
2. Device Integration and Intelligence: Future work should aim to create smart, all-in-one sensor chips. These chips would combine tiny CLCs sensors with cameras and artificial intelligence (AI) software. The AI would be trained to automatically interpret complex optical changes from the CLCs, such as texture shifts or iridescence, converting them into clear, digital results. This makes the system more robust against interference, reduces human error in reading the signals, and provides trustworthy answers.
3. Modeling and Computational Analysis: Developing a quantitative model that links stimulus, molecular rearrangement, and optical response is crucial. The integration of computational approaches such as Landau–de Gennes theory with high-resolution imaging will enable a deeper understanding and better control of defect and pattern behavior in curved 3D structures.
4. External Field Regulation and Integration Technology: In the future, integrating external fields such as acoustic, optical, electrical, and magnetic fields to actively manipulate CLCs is an important research direction. On the one hand, CLCs can couple with electromagnetic or optical fields through mechanisms such as dielectric anisotropy, magnetic anisotropy, and photo-isomerization (e.g., azobenzene) to precisely regulate molecular alignment [19]. On the other hand, external-field manipulation has shown promise in droplet microfluidics [120,121]. For instance, surface acoustic waves enable contact-free droplet operations (merging, splitting, mixing) while supporting microchannel cleaning and interface resetting. Hence, the introduction of external fields (e.g., light, electricity, sound) without disrupting the soft CLC architecture can actively enhance mass transfer and interfacial dynamics, facilitating rapid and uniform analyte transport, as well as interface refreshing and resetting [122]. Meanwhile, advanced heterogeneous integration processes should be developed to realize integrated drive, sensing, and readout systems.

In summary, CLCs uniquely convert molecular information into macroscopic optical signals. Their combination with advanced confinement and microfluidic-assisted confinement strategies makes them a key candidate for driving innovation in biomedicine, environmental monitoring, and industrial applications. While challenges in stability, signal interpretation, and system integration remain, future progress is anticipated through several focused efforts. These include the co-optimization of geometric parameters, the adoption of intelligent AI-based readout technologies, and the application of active external field modulation. Together, these advances are expected to enable coordinated breakthroughs in material design, signal analysis, and system integration, ultimately guiding CLC-based sensing technologies toward practical and mature application.

## Figures and Tables

**Table 1:** Dimensionless Numbers Relevant to Droplet Generation.

Dimensionless Constant	Formula	Physical Meaning
Re	$Re=\frac{\rho vL}{\eta }$	Inertial force/Viscous force
Ca	$Ca=\frac{\eta v}{\sigma }$	Viscous force/Interfacial tension
We	$We=\frac{\rho v^{2}L}{\sigma }$	Inertial force/Interfacial tension
Bo	$Bo=\frac{\Delta g\rho L^{2}}{v^{2}}$	Gravitational force/Interfacial tension

Notes: η is the viscosity of the continuous phase in Pa·s, v is the velocity of the continuous phase in m/s, σ is the interfacial tension in N/m, ρ is the fluid density in kg/m^3^, L is the length of the microchannel in m, and g is the gravitational acceleration in m/s^2^.

**Table 2:** Summarization of 3D-confined CLC sensors.

CLC Description	3D Structure	Fabrication Route of 3D Structure	Dimensional Parameters	Anchoring State/Optical State	Target	Readout Method and Geometric Conditions	Sensing Performance	Ref.
BHR-59001 + S-811; Non-polymerizable	Microcapsule	Capillary device; Double emulsion; PEGDA hydrogel shell cross-linking stabilization; PVA/Pluronic F108 interfacial anchoring	d = 80–140 μm; δ = 13.3 μm; CV: 1.71–1.89%	Planar anchoring; Helical texture, reflected light is circularly polarized light	Temperature	Spectrometry; No fixed incident angle; No polarization	Sensitivity: 1.40 nm/°C; Linear range: 56.9–80 °C; RT: 3 min (equilibration time, 20–88 °C)	[117]
ZLI-2293 + MLC-6248; Non-polymerizable	Microcapsule	Capillary device; Double emulsion; PVA interfacial stabilization; Tangential anchoring	d = 341 μm; δ = 25 μm	Tangential anchoring; Helical axis perpendicular to the surface; Omnidirectional photonic structure	H_2_O_2_	Spectrometry; Collected perpendicular to the reflection surface; No polarization	Sensitivity: 101 V/mm^3^; RT: 50 s (linear rise time); LOD: Not reported	[118]
MLC-2132 + CB15; Non-polymerizable	Droplet	PDMS flow-focusing device; Single emulsion; PAA-b-LCP block copolymer stabilization; pH-responsive anchoring transition (protonation/deprotonation)	Dimensional parameters: Not reported	Tangential/perpendicular anchoring; Frank-Pryce spherulitic texture; Central reflection spot	Glucose; Cholesterol	Imaging method; Reflection mode; No polarization	Glucose: LOD = 0.5 μM, RT: ≤4 s; Cholesterol: LOD = 2.5 μM, RT: ≤4 s	[104]
CH100-650/CH100-550/CH100-450 (No explicit host + chiral dopant); Non-polymerizable	Microcapsule	Capillary device; Single emulsion; Polyurethane shell interfacial polymerization stabilization; PVA interfacial anchoring	d = 110–160 μm; δ = 400–650 nm; CV: Narrow distribution (Not reported in literature)	Planar anchoring; Radial helical axis, Concentric ring texture	Temperature	Imaging method; Reflection mode; No polarization	Linear range: Red at 67 °C, Green at 49 °C, Blue at 58 °C; RT: Reversible switching (25–67 °C cycles)	[30]
RMM727 + CB15; Polymerizable (RMM727 is reactive mesogen)	Shell	Capillary device; Emulsion type not specified; IPN stabilization; PVA interfacial anchoring	d = 100–155 μm; δ = 6–19 μm	Planar anchoring; Helical texture, Central reflection spot	Acetone; THF; Pyridine; Acrylic acid	Spectrometry; Reflection mode; No polarization	Semi-quantitative analysis; RT: Not reported	[98]
RMM727 + CB15; Polymerizable (RMM727 is reactive mesogen)	Solid particle	PDMS flow-focusing device; Single emulsion; PAA network IPN stabilization; pH-responsive anchoring	d = 80 μm; Shell thickness: Not reported	Planar/homeotropic anchoring (pH-regulated); Helical texture; Reflected color changes with swelling	pH; Divalent metal ions (Ca^2+^, Mg^2+^); Urea; Glucose	Imaging method; Reflection mode; No polarization	Urea: LOD = 0.027 mM, Linear range: 0–7.5 mM; Divalent ions: LOD = 9 μM, Linear range: 0–0.4 mM; Glucose: Visual detection upper limit 20 mM; RT: not reported	[107]
COC + Unspecified host (nCB series); Non-polymerizable	Microcapsule	Capillary device; Double emulsion; Hydrogel shell stabilization; PVA interfacial anchoring	d = 132 μm; δ = 10.5 μm	Planar anchoring; Helical texture, Temperature-dependent reflected color	Temperature	Spectrometry; No fixed incident angle; No polarization	Sensitivity: > 105 nm/°C; Linear range: 2–37 °C; RT: Heating 26 s, Cooling 85 s	[97]
E7 + CB15; Non-polymerizable	Janus droplet	Microfluidic type not specified; Double emulsion; IgG functionalization stabilization; Antigen–antibody competitive binding-regulated anchoring	Dimensional parameters: Not reported	Homeotropic anchoring; Helical pitch changes with binding interaction	Salmonella	Imaging method; Reflection mode; No polarization	LOD = 10^3^–10^4^ cells/mL; RT: ~3 h (room temperature, pH 7.2)	[115]
RMM727 + CB15; Polymerizable (RMM727 is reactive mesogen)	Solid particle	PDMS flow-focusing device; Single emulsion; PAA network IPN stabilization; K+ ion pre-modification anchoring	Dimensional parameters: Not reported	Homeotropic anchoring; Helical texture, Metal ion-induced bridging structure	Urea; Ca^2+^	Spectrometry; Reflection mode; No polarization	Urea: LOD = 1.52 mM, Linear range 3.5–14 mM; Ca^2+^: LOD = 0.09 mM, Linear range 0–0.4 mM; RT: 3 min (equilibration time)	[96]
E7 + R5011; Non-polymerizable	Droplet	Capillary flow-focusing device; Single emulsion; PVA/SC12S mixed stabilization; Competitive adsorption-regulated anchoring transition	d = 40–80 μm; Shell thickness: Not reported; Monodispersity: Excellent	MonodispersityHomeotropic→planar anchoring (bile acid-induced); Flashing spot→central reflection spot transition	CA; DCA	Imaging method; Reflection mode; No polarization	CA: LOD = 1 μM; DCA: LOD = 0.5 μM; RT: 40–53 s (pH 7.2, 80 μm droplet)	[114]
5CB + RM257 + LC756; Polymerizable (RM257 is reactive mesogen)	Droplet	Capillary device; Single emulsion; PVA stabilization; Planar anchoring, birefringence effect regulation	d = 92–149 μm; Shell thickness: Not reported; CV: Narrow distribution (Not reported in original literature)	Planar anchoring; Central + brush-like dual-structure texture, enhanced birefringence	TFA; Ethanol; Methanol	Spectrometry + Imaging method; No fixed incident angle; Involving polarization	Semi-quantitative analysis; RT: 330–400 s; LOD: Not reported	[116]
E7 + R-5011; Non-polymerizable	Droplet	Capillary flow-focusing device; Single emulsion; PVA//SC12S mixed stabilization; Electrostatic adsorption-regulated anchoring	d = 100 ± 10 μm; Shell thickness: Not reported; Monodispersity: Excellent	Homeotropic→planar anchoring (BSA adsorption-induced); Flashing spot→central reflection spot transition	BSA	Imaging method; Reflection mode; No polarization	LOD = 0.15 μM (pH 2–4), 0.30 μM (pH 5); RT: 159 s (0.37 μM BSA, pH 4)	[119]
nCB (n = 4–8) + COC; Non-polymerizable	Droplet	PDMS microfluidic device; Single emulsion; PVA/SDS stabilization; Surfactant-induced planar anchoring	d = 100 ± 10 μm; Shell thickness: Not reported; CV < 10%	Planar anchoring; Radial helical axis, concentric ring texture	Temperature	Spectrometry; Fixed incident angle; No polarization	Sensitivity: >100 nm/°C; Linear range: 10–40 °C; RT: Hysteresis-free (heating/cooling cycles)	[12]

Abbreviation definition: d = Diameter of 3D confined CLC structure; δ = Shell thickness of microcapsule/shell structure; CV = Coefficient of variation; PEGDA = Poly(ethylene glycol) diacrylate. Notes: LOD: Refers to the minimum concentration/content of the target analyte at which the sensor produces a distinguishable signal (spectral shift, intensity change, or texture transition). Sensitivity: Defined as the magnitude of signal change induced by a unit concentration/content of the target analyte. For spectral-based sensors, the quantitative standard is “signal wavelength shift/analyte concentration” (e.g., nm/°C, nm/mM). For texture transition-based sensors, it is characterized by “the minimum change in analyte concentration corresponding to a distinguishable signal”. Linear range: Refers to the interval where the sensor signal exhibits a linear correlation with the concentration/content of the target analyte. RT: Uniformly defined as “the time required from the contact between the target analyte and the sensor until the signal reaches a stable value”. Distinctions are clearly made between terms such as “equilibration time” and “linear rise time”, and the original time definitions from the literature (e.g., “equilibration time”, “linear rise time”) have been supplemented in the table.

## Data Availability

No new data were created or analyzed in this study.
